# Guidelines and Standard Frameworks for AI in Medicine: Protocol for a Systematic Literature Review

**DOI:** 10.2196/47105

**Published:** 2023-10-25

**Authors:** Kirubel Biruk Shiferaw, Moritz Roloff, Dagmar Waltemath, Atinkut Alamirrew Zeleke

**Affiliations:** 1 Department of Medical Informatics Institute for Community Medicine University Medicine Greifswald Greifswald Germany

**Keywords:** artificial intelligence, biomedical, guidelines, machine learning, medicine

## Abstract

**Background:**

Applications of artificial intelligence (AI) are pervasive in modern biomedical science. In fact, research results suggesting algorithms and AI models for different target diseases and conditions are continuously increasing. While this situation undoubtedly improves the outcome of AI models, health care providers are increasingly unsure which AI model to use due to multiple alternatives for a specific target and the “black box” nature of AI. Moreover, the fact that studies rarely use guidelines in developing and reporting AI models poses additional challenges in trusting and adapting models for practical implementation.

**Objective:**

This review protocol describes the planned steps and methods for a review of the synthesized evidence regarding the quality of available guidelines and frameworks to facilitate AI applications in medicine.

**Methods:**

We will commence a systematic literature search using medical subject headings terms for medicine, guidelines, and machine learning (ML). All available guidelines, standard frameworks, best practices, checklists, and recommendations will be included, irrespective of the study design. The search will be conducted on web-based repositories such as PubMed, Web of Science, and the EQUATOR (Enhancing the Quality and Transparency of Health Research) network. After removing duplicate results, a preliminary scan for titles will be done by 2 reviewers. After the first scan, the reviewers will rescan the selected literature for abstract review, and any incongruities about whether to include the article for full-text review or not will be resolved by the third and fourth reviewer based on the predefined criteria. A Google Scholar (Google LLC) search will also be performed to identify gray literature. The quality of identified guidelines will be evaluated using the Appraisal of Guidelines, Research, and Evaluation (AGREE II) tool. A descriptive summary and narrative synthesis will be carried out, and the details of critical appraisal and subgroup synthesis findings will be presented.

**Results:**

The results will be reported using the PRISMA (Preferred Reporting Items for Systematic Review and Meta-Analyses) reporting guidelines. Data analysis is currently underway, and we anticipate finalizing the review by November 2023.

**Conclusions:**

Guidelines and recommended frameworks for developing, reporting, and implementing AI studies have been developed by different experts to facilitate the reliable assessment of validity and consistent interpretation of ML models for medical applications. We postulate that a guideline supports the assessment of an ML model only if the quality and reliability of the guideline are high. Assessing the quality and aspects of available guidelines, recommendations, checklists, and frameworks—as will be done in the proposed review—will provide comprehensive insights into current gaps and help to formulate future research directions.

**International Registered Report Identifier (IRRID):**

DERR1-10.2196/47105

## Introduction

### Rationale

Artificial intelligence (AI) has become a hot topic in biomedical and clinical routines in the past decade [1,2]. In fact, an increasing number of scientific publications suggest algorithms and AI models for target diseases and conditions [3]. The application of AI in health care ranges from medical image analysis to text mining, targeting prognosis, diagnosis, event and outcome prediction, and treatment [1]. The number of scientific contributions in peer-reviewed journals focusing on AI applications in medicine is also highly increasing [4,5].

The comparison of algorithm performance for a target condition has become a common way of presenting and evaluating machine learning (ML) models. The diversity of ML models leads to competition and fast progress. However, the current situation leaves health care providers unsure about which ML model to use given multiple alternative models for a specific target [6] and the “black box” nature of ML [7]. Moreover, the fact that studies do not use guidelines consistently in developing and reporting AI models poses another challenge in trusting and adapting models for practical implementation [8-10].

In fact, guidelines and recommended frameworks for developing and reporting AI studies have been published by different experts and work groups to facilitate reliable assessment of model validity and consistent interpretation [11-14]. In addition, the authors have proposed recommendations for the evaluation of AI studies [15-18]. This review protocol describes the planned steps and methods of a future review that aims to synthesize evidence regarding the quality of available guidelines and frameworks developed to facilitate AI applications in medicine and summarize their content.

### Objective

The systematic review will address the following scientific questions:

- What are the available guidelines and frameworks with regard to predictive AI model development and reporting in medicine?
- What are the main aspects (target or purpose) of the available guidelines and frameworks?
- What is the quality of the available guidelines with respect to the Appraisal of Guidelines, Research and Evaluation (AGREE II) domains?
- What are the reported implementation challenges in the available guidelines?

## Methods

### Eligibility Criteria

All guidelines, standard frameworks, best practices, checklists, and recommendations will be included, irrespective of the study design. Studies will be limited to English and to the time period between database inception and June 2023 (the time of data collection).

### Search Strategy and Information Sources

This protocol adheres to the PRISMA-P (Preferred Reporting Items for Systematic Review and Meta-Analysis Protocols) 2015 [19]. A systematic literature search will be commenced using Medical Subject Headings (MeSH) terms and keywords for medicine, guidelines, and ML (Table S1 in Multimedia Appendix 1). The initial search will be conducted on 3 web-based repositories: PubMed, Web of Science, and EQUATOR (Enhancing the Quality and Transparency of Health Research). The EQUATOR network is a global initiative working toward improving the value of research by promoting robust reporting guidelines [20]. A Google Scholar search will also be performed to identify gray literature. References in selected literature will be scanned, and relevant papers will be included after discussion among the reviewers.

After the comprehensive search, all bibliographic information will be uploaded to a web-based systematic review tool (Rayyan) and afterward processed with CADIMA [21] for further screening and preliminary analysis.

### Study Selection

After removing duplicate results, a preliminary scan for titles will be done by 2 reviewers. After the initial scan, the reviewers will rescan the selected literature for abstract review, and any incongruities about whether to include the article for full-text review will be resolved by the third reviewer based on the predefined criteria.

### Data Extraction, Collection, and Management

Once the selection process has been finalized, relevant information from the selected literature will be extracted by the 2 reviewers independently using a predefined information extraction sheet. At this stage, discrepancies will be resolved by the third and fourth reviewers.

The information extraction sheet will be designed to collect relevant information such as study characteristics (authors, year of publication, study type, aspect, specific disease or condition focused, and standard followed) from selected literature for further synthesis. “Study type” in this context refers to whether the study is a guideline, a framework, a suggestion, a checklist, a best practice, or a recommendation. The term “aspect” entails the purpose for which the guideline or the framework is primarily designed. Guidelines or frameworks could be designed for different purposes. For instance, if a guideline is developed to elaborate on or declare how ML studies should report their findings, the aspect or purpose will be a “reporting aspect.” If, however, the guideline emphasizes the ethical dimension of ML in medicine, the aspect will be referred to as “ethical aspect.” A study can have multiple aspects (sometimes a guideline or a framework could be about both the development and reporting procedures and sometimes only the reporting or the development procedure), and it will also be extracted under the “aspect” category. The column “specific disease or condition focused” refers to whether the guideline or framework is subject to or validated for a specific disease or condition. Sometimes, researchers suggest customized and focused guidelines for a specific condition. Based on the predefined data extraction Excel (Microsoft Corp) sheet, 2 reviewers will extract the data independently, and any sort of discrepancy will be discussed with the 3rd and 4th reviewers after every 10 extractions. The data extraction template is presented in Table 1.

**Table 1:** Data extraction template.

Sections			Description
**Section 1: study characteristics**			
	First author’s name	For example, John Smith	
	Year of publication	For example, YYYY	
	Title	The title of the study	
	Journal	The name of the journal. For example, scientific reports	
	Country of first author	For example, United States of America	
**Section 2: research questions**			
	Type of outcome	Provide a description about the study whether it is a guideline, standard framework, recommendation, checklist, best practice, or expert opinion.	
	Aspect	Provide the primary purpose for which the guideline or the framework is designed. For example, reporting, development, ethics, and governance.	
	Standard followed	Provide a description of the standard followed during the development of the proposed guideline or framework. For example, the EQUATOR^a^ network.	
	Guidelines and frameworks with regard to predictive artificial intelligence model development and reporting in medicine	Provide a description of the content and domain of the guideline or framework.	
	The quality of the available guidelines	Provide a quantified quality assessment of the identified guidelines based on AGREE II^b^ assessment.	
	Reported gaps in the available guidelines	Describe the reported gaps and implementation challenges.	
	Specific target domain	Provide information if the guideline or framework is designed for a specific disease domain.	

^a^EQUATOR: Enhancing the Quality and Transparency of Health Research.

^b^AGREE II: Appraisal of Guidelines, Research, and Evaluation.

### Quality and Risk of Bias Assessment

The quality of identified guidelines will be evaluated using the AGREE II tool [22]. AGREE II assesses the quality of guidelines in terms of the methodological rigorousness and transparency of the guideline development process [22]. It consists of 23 key items organized within 6 domains (scope and purpose, stakeholder involvement, rigor of development, clarity of presentation, applicability, and editorial independence) and 2 global rating items for overall assessment. As recommended by the AGREE II user manual, 4 appraisers will individually appraise the selected guidelines and frameworks, and the percentage of quality will be calculated based on the ratings of each quality domain [22]. Other frameworks and best practices proposed by researchers will be systematically summarized.

### Analysis

A descriptive summary and narrative synthesis will be carried out, and the details of critical appraisal and subgroup synthesis findings will be presented. This systematic review will present the list of available guidelines, frameworks, suggestions, checklists, best practices, or recommendations for developing and reporting AI studies in medicine and assess the quality of the guidelines and discuss the aspects.

## Results

We anticipate finishing the systematic literature review by November 2023. The expected result from this review will explore and highlight the quality of the available guidelines and point out the existing gaps. Furthermore, the synthesis from the checklists and recommendations will provide a comprehensive set of standardized tools.

## Discussion

Guidelines are important sets of instructions that facilitate transparent and reproducible scientific processes [23]. The potential benefits of guidelines are, however, only as good as the quality of the guidelines themselves. This protocol describes the setup of a systematic review to identify available guidelines, frameworks, best practices, and recommendations for developing and reporting AI studies in medicine. The review will assess the quality of guidelines, summarize the gap in guidelines, and identify critical considerations in the application of AI in medicine. As the search is limited to English due to resource limitations, there might be language bias, indicating that some important studies conducted in different languages might be missed. Thus, conclusions should be drawn carefully.

## Appendix

Multimedia Appendix 1 Keywords.

## Abbreviations

AGREE II: Appraisal of Guidelines, Research and Evaluation
AI: artificial intelligence
EQUATOR: Enhancing the QUAlity and Transparency Of health Research
MeSH: medical subject headings
ML: machine learning
PRISMA: Preferred Reporting Items for Systematic Review and Meta-Analyses
PRISMA-P: Preferred Reporting Items for Systematic Review and Meta-Analysis Protocols
