# Supplementary material for: Guidelines and Standard Frameworks for AI in Medicine: Protocol for a Systematic Literature Review
Source: JMIR Res Protoc. 2023 Oct 25;12:e47105. doi: 10.2196/47105 (PMC10632920; doi:10.2196/47105)
Supplement: Multimedia Appendix 1 [file resprot_v12i1e47105_app1.docx]

Multimedia Appendix 1

Table S1: Keywords for Guidelines and Standard Frameworks for Artificial Intelligence in Medicine

| **No.** | **Keywords for AI** | **No.** | **Search Iteration** |
| --- | --- | --- | --- |
| **1** | Machine Learning |  |  |
| 2 | Artificial intelligence | 25 | 1 OR 2 OR 3 OR 4 |
| 3 | deep learning | 26 | 5 OR 6 OR 7 OR 8 OR 9 OR 10-16 |
| 4 | Natural language processing | 27 | 25 AND 26 |
|  | **Keywords for guidelines** | 28 | 17 OR 18 OR 19 OR 20-24 |
| 5 | Guideline* | 29 | 27 AND 28 |
| 6 | standard* |  |  |
| 7 | framework* |  |  |
| 8 | best practice* |  |  |
| 9 | Reporting |  |  |
| 10 | Protocol |  |  |
| 11 | reporting guideline |  |  |
| 12 | quality assessment |  |  |
| 13 | Checklist |  |  |
| 14 | recommend* |  |  |
| 15 | suggest* |  |  |
| 16 | prefer* |  |  |
|  | **Keywords for medicine** |  |  |
| 17 | Health* |  |  |
| 18 | Health science |  |  |
| 19 | Health Service |  |  |
| 20 | medicine |  |  |
| 21 | disease* |  |  |
| 22 | Healthcare |  |  |
| 23 | Biomedicine |  |  |
| 24 | Biomedical science |  |  |
